# Effects of inhaled nitric oxide for postoperative hypoxemia in acute type A aortic dissection: a retrospective observational study

**DOI:** 10.1186/s13019-020-1069-6

**Published:** 2020-01-22

**Authors:** Hang Zhang, Yaoyang Liu, Xiangdong Meng, Dicheng Yang, Sheng Shi, Jian Liu, Zhongxiang Yuan, Tongtong Gu, Lin Han, Fanglin Lu, Zhiyun Xu, Yang Liu, Min Yu

**Affiliations:** 1 Department of Cardiovascular Surgery, Shanghai General Hospital, Shanghai Jiao Tong University School of Medicine, No. 100 Haining Road, Shanghai, 200080 China; 2 Department of Cardiovascular Surgery, Shanghai General Hospital, Nanjing Medical University, Shanghai, 200080 China; 3 Department of Rheumatology and Immunology, Changzheng Hospital, Second Military Medical University, 415 Fengyang Road, Shanghai, 200000 China; 4 Department of Central laboratory, Nanjing First People’s Hospital, Nanjing Medical University, NO.68 Changle Road, Nanjing, 210006 China; 5 Department of Cardiovascular Surgery, Changhai Hospital, Second Military Medical University, Shanghai, 200433 China; 6 Department of Critical Care Medicine, Naval medical Center of PLA, Shanghai, 200433 China

**Keywords:** Postoperative hypoxemia, Acute type A aortic dissection, Inhaled nitric oxide, Propensity analysis

## Abstract

**Background:**

Postoperative hypoxemia in acute type A aortic dissection (AADA) is a common complication and is associated with negative outcomes. This study aimed to analyze the efficacy of low-dose (5–10 ppm) inhaled nitric oxide (iNO) in the management of hypoxemia after AADA surgery.

**Methods:**

In this retrospective observational study, Medical records of patients who underwent AADA surgery at two institutions between January 2015 and January 2018 were collected. Patients with postoperative hypoxemia were classified as iNO and control groups. Clinical characteristics and outcomes were compared using a propensity score-matched (PSM) analysis.

**Results:**

Among 436 patients who underwent surgical repair, 187 (42.9%) had hypoxemia and 43 were treated with low-dose iNO. After PSM, patients were included in the iNO treatment (*n* = 40) and PSM control (*n* = 94) groups in a 1:3 ratio. iNO ameliorated hypoxemia at 6, 24, 48, and 72 h after initiation, and shortened the durations of ventilator support (39.0 h (31.3–47.8) vs. 69.0 h (47.8–110.3), *p* < 0.001) and ICU stay (122.0 h (80.8–155.0) vs 179.5 h (114.0–258.0), *p* < 0.001). There were no significant between-group differences in mortality, complications, or length of hospital stay.

**Conclusions:**

In this study, we found that low-dose iNO improved oxygenation in patients with hypoxemia after AADA surgery and shortened the durations of mechanical ventilation and ICU stay. No significant side effects or increase in postoperative mortality or morbidities were observed with iNO treatment. These findings warrant a randomized multicenter controlled trial to assess the exact efficiency of iNO for hypoxemia after AADA.

## Introduction

Acute type A aortic dissection (AADA) is a fatal condition. The mortality rate is 1–2% per hour on the first day, with nearly 50% of deaths occurring within the first week [1, 2]. Surgery is life-saving for most patients but may result in high postoperative morbidity [1, 2].

Postoperative hypoxemia is a serious complication with an incidence of 30–50% [3, 4]. Hypoxemia occurs secondary to systemic and local inflammatory reactions after aortic vascular tissue destruction, ischemic/reperfusion injury, intraoperative cardiopulmonary bypass (CPB), deep hypothermia, and massive blood transfusion [4–7]. Hypoxic pulmonary vasoconstriction is inhibited, resulting in ventilation/perfusion mismatching and shunting [7]. Hypoxemia prolongs postoperative mechanical ventilation and intensive care unit (ICU) stay and increases postoperative mortality. However, effective medical interventions are limited and controversial [6–8].

Inhaled nitric oxide (iNO) is a selective pulmonary vasodilator that has long been used in the management of acute respiratory distress syndrome (ARDS), pulmonary hypertension, neonatal hypoxemic respiratory failure, and lung transplantations. To date, several randomized controlled trials and meta-analyses have concluded that iNO therapy wasn’t beneficial to mortality or mechanical ventilation duration of patients with ARDS [9, 10]. However, postoperative hypoxemia in AADA was different with ARDS in etiology and pathophysiology. To the best our knowledge, no randomized controlled or case-control study has evaluated iNO treatment efficacy to this group of patients.

We previously found that iNO improved oxygenation after AADA and tended to decrease the time to extubation [11]. Herein, we retrospectively analyzed the effects of low-dose iNO therapy in patients with postoperative hypoxemia after AADA and evaluated its efficacy and safety.

## Patients and methods

### Patients

We retrospectively reviewed consecutive patients with AADA who underwent surgical repair from January 2015 to January 2018 at Shanghai General Hospital and Changhai Hospital.

**Fig. 1:**
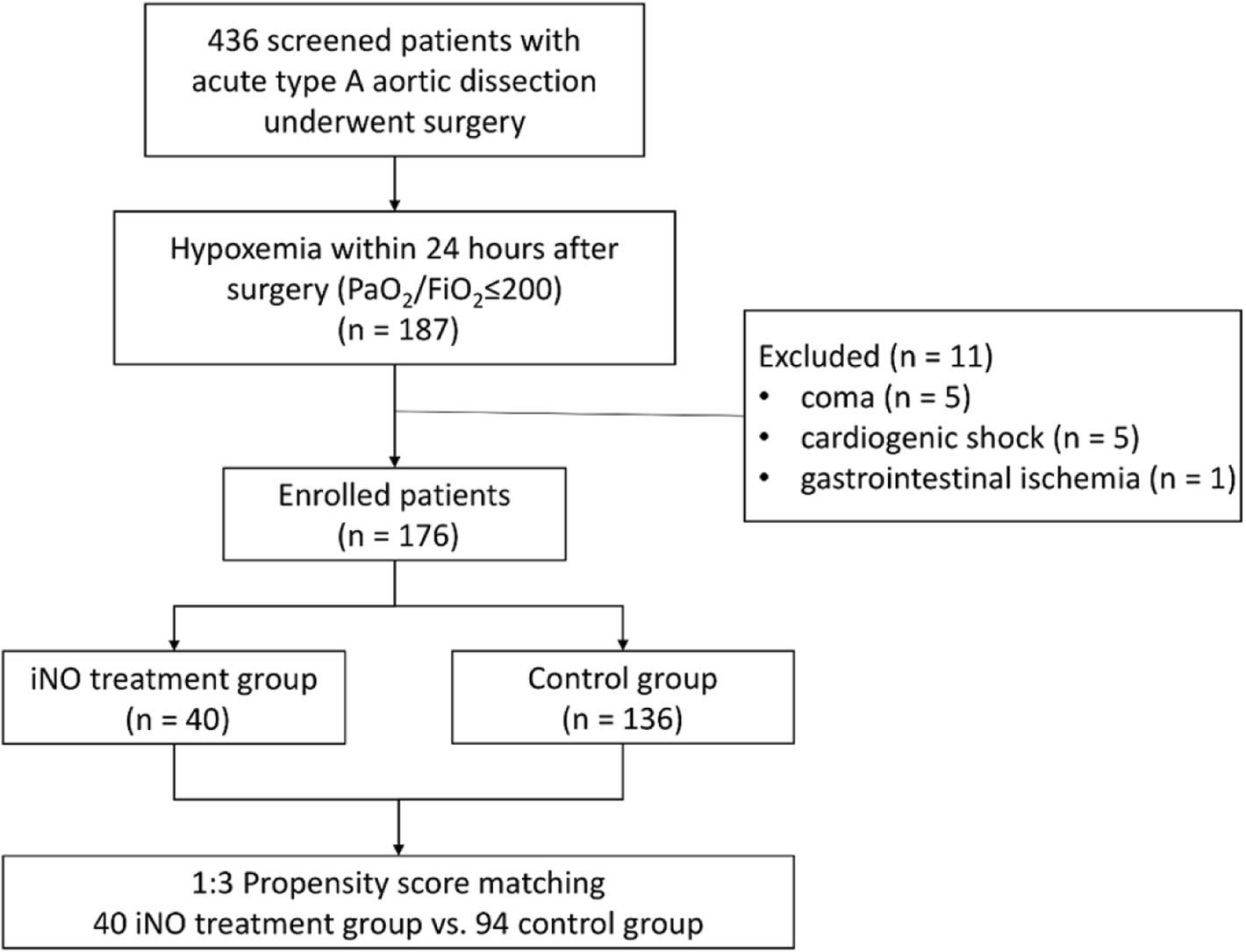
Flowchart showing patients included in the analysis. iNO: inhaled nitric oxide

The enrollment criteria were as follows: (I) patients who received repairment surgery for AADA; (II) patients with a persistent postoperative hypoxemia, which was defined as the blood gas exam showed that ratio of arterial partial pressure of oxygen (PaO_2_) to fraction of inspired oxygen (FiO_2_) was equal to or less than 200 mmHg (PaO_2_/FiO_2_ ≤ 200) occurring within 24 h after ICU admission, lasting more than 2 h, and in the absence of other causes of pulmonary insufficiency such as cardiogenic pulmonary edema, pneumonia, pleural effusion, segmental atelectasis, pneumothorax, and pulmonary artery embolism [12]. The exclusion criteria were as follows: (I) patients who died within 24 h after surgery; (II) patients who developed severe postoperative complications such as: coma, cardiogenic shock, and gastrointestinal ischemia. Eligible patients were divided into two groups: patients who received iNO (iNO group) and patients who were managed routinely (control group) (Fig. 1).

This study was approved by the Ethics Committee of Shanghai General Hospital, Shanghai Jiao Tong University School of Medicine, (No. 2018KY241) and the Committee on Ethics of Biomedicine Research, Second Military Medical University, Shanghai (No. SMMUEC2018–044), with the need for individual patient consent waived.

### Surgical procedures and standard postoperative ICU protocols

All enrolled patients underwent aortic repair under extracorporeal circulation, deep hypothermic circulatory arrest, and selective cerebral perfusion. Repairment surgery for AADA was performed with the same standard surgical procedure. The surgical approaches are shown in Table 1. The standard ICU protocols were approximately similar in both hospitals and included ventilator support, sedation, and pain management. Briefly, patients were placed on ventilators in synchronized intermittent mandatory ventilation (SIMV) or assist/control (A/C) modes set at 8–10 mL/kg tidal volume and 5 cmH_2_O positive end-expiratory pressure (PEEP). Arterial blood gases (ABG) were check every 30 min to 6 h, depending on the patient’s condition. Propofol at 20–50 μg/kg/min was administered until the patient was awake. A continuous infusion of a small dose of fentanyl was administered until ventilator weaning. Routine management for postoperative hypoxemia included: (1) recruitment maneuvers with PEEP of 10–15 cm H_2_O; (2) negative fluid balance maintenance if hemodynamic stability could be achieved; (3) administration of methylprednisolone 40 mg and ulinastatin 300,000 units intravenously twice daily for 3 days; (4) bilevel positive airway pressure (BiPAP) noninvasive ventilation support after extubation, when necessary.

**Table 1:** Baseline characteristics of patients with AADA and hypoxemia within 24 h after surgery: pre-PSM

Variable	Control (*N* = 136)	iNO treatment (*N* = 40)	*p*-value
Age, y	50.4 ± 11.0	49.9 ± 11.2	0.789
Men	110 (80.9)	31 (77.5)	0.638
BMI	26.5 ± 2.7	26.3 ± 2.9	0.707
Comorbidity			
Hypertension	96 (70.6)	32 (80.0)	0.240
Diabetes	2 (1.5)	2 (5.0)	0.476
COPD	3 (2.2)	1 (2.5)	1.000
Hepatic insufficiency	4 (2.9)	1 (2.5)	1.000
Renal insufficiency	27 (19.9)	12 (30.0)	0.174
Cerebrovascular event	3 (2.2)	2 (5.0)	0.694
Myocardial infarction	9 (6.6)	2 (5.0)	1.000
tamponade	21 (15.4)	9 (22.5)	0.297
Marfan Syndrome	6 (4.4)	3 (7.5)	0.711
Smoking	32 (23.5)	8 (20.0)	0.640
Laboratory test			
Leucocyte (10^9^/L)	12.0 ± 4.0	13.0 ± 3.4	0.147
Hemoglobin (g/L)	132.0 (123.0, 143.0)	137.5 (124.8, 150.3)	0.112
Platelet (10^9^/L)	147.0 (121.3, 201.8)	161.5 (140.5, 200.0)	0.317
ALT (U/L)	26.5 (19.3, 38.0)	30.5 (19.0, 41.4)	0.930
Creatinine (μmol/L)	85.0 (68.0, 107.8)	89.5 (70.3, 132.0)	0.437
Troponin I (ng/ml)	0.02 (0.01, 0.05)	0.02 (0.01, 0.09)	0.782
PaO_2_ (mmHg)	85.5(71.2, 101.7)	80.0(67.7, 109.2)	0.897
Surgical procedure			
Total arch replacement	129 (94.9)	38 (95.0)	1.000
Hemi arch replacement	7 (5.1)	2 (5.0)	1.000
Concomitant CABG	10 (7.4)	2 (5.0)	1.000
CPB			
CPB time (min)	167.5 (143.5, 186.8)	162.5 (147.0, 191.5)	0.806
ACC time (min)	97.5 (84.0, 109.8)	96.0 (87.3, 117.3)	0.604
DHCA time (min)	28.0 (22.0, 31.8)	24.0 (19.0, 28.0)	0.041*
Minimum temperature in CPB (°C)	22.3 (22.1, 24.4)	22.1 (21.5, 23.5)	0.040*
PaO_2_/FiO_2_ ratio meet hypoxemia criteria	112.9 (94.3, 155.0)	95.0 (82.9, 127.1)	0.076

*AADA* Acute type A aortic dissection, *PSM* Propensity score-matching, *iNO* Inhaled nitric oxide, *BMI* Body mass index, *COPD* Chronic obstructive pulmonary disease, *ALT* Alanine transaminase, *CABG* Coronary artery bypass graft, *CPB* Cardiopulmonary bypass, *ACC* Aortic cross-clamp, *DHCA* Deep hypothermic circulatory arrest, *PaO*_*2*_ Partial pressure of oxygen, *FiO*_*2*_ Fraction of inspired oxygen

*p < 0.05

Patients were extubated after meeting the following criteria: (1) alert and cooperative with adequate muscle strength; (2) hemodynamically stable with no signs of low cardiac output syndrome or myocardial ischemia, and minimal need for inotropic support (noradrenaline or adrenaline ≤0.05 μg/kg/min; (3) chest tube drainage < 50 mL/h with no active bleeding; (4) acceptable ABG at FiO_2_ ≤ 0.5 and PEEP ≤5 cmH_2_O of PaO_2_ ≥ 80 mmHg, and PaCO_2_ < 45 mmHg, in the absence of respiratory distress. Patients were transferred out of the ICU when hemodynamically stable, without serious complications, and with PaO_2_ ≥ 80 mmHg during O_2_ administration via Venturi mask or nasal cannula.

### iNO management

At our institutes, iNO treatment is optional for symptomatic treatment of hypoxemia. Thus, iNO is administered at the discretion of the intensivist. A number of patients who met the criteria for hypoxemia received low dose iNO (5–10 ppm [ppm]) in addition to routine management. NO gases and equipment were supplied by the Children’s Hospital of Fudan University, as previously described [13]. Briefly, the NO inhalation device was managed by a flow controller (MFC) (Shanghai Noventek, Shanghai, China). The N_2_-based gas mixture flowed into the breathing circuit. The NO/NO_2_ electrochemical sensor (NOxBOX Plus®; Bedfont Scientific, Rochester, England) was placed in the breathing circuit near the intubation cannula. Continuous monitoring was instituted to maintain the iNO concentration at 5–10 ppm and NO_2_ at less than 3 ppm. Methemoglobin (MetHb) levels were measured once daily using the Radical-7 pulse oximeter (Radical-7® Pulse CO-Oximeter®, Masimo, USA). Side effects included increasing pleural drainage, new-onset bleeding, and thrombocytopenia (platelet count < 500,000/ul). If oxygenation did not improve within 24 h, if any side effects were noted, or if abnormal methemoglobin and NO_2_ were observed, iNO therapy was discontinued. When the patient was extubated, iNO was administered via nasal cannula and weaned by decreasing the flow gradually within 24 h.

### Outcomes

The primary outcomes were: (1) PaO_2_/FiO_2_ collected at 7 time points: preoperative; postoperative ICU admission; first episode of hypoxemia; 4–6 h after iNO initiation (iNO group) or 4–6 h after hypoxemia onset (control group); 24, 48, and 72 h postoperatively; and (2) duration of mechanical ventilation and length of ICU stay. Secondary outcomes included mortality, complications, and in-hospital stay. Possible iNO related side-effects such as pleural drainage and thrombocytopenia were also observed. Coma was defined by a complete absence of consciousness with computed tomography (CT)-proven cerebrovascular occlusion. Cardiogenic shock was diagnosed in the presence of sustained hypotension (systolic blood pressure < 90 mmHg) and low cardiac output, with a poor response to high-dose inotropes and vasopressors. Gastrointestinal ischemia was defined by clinical symptoms and CT angiography evidence of deficient gastrointestinal blood supply with mesenteric artery involvement. Renal insufficiency was defined by an estimated glomerular filtration rate (eGFR) < 60 ml/min/1.73 m^2^; acute kidney injury (AKI) was defined by either a 50% increase from baseline in serum creatinine within 7 days after surgery, or a 0.3 mg/dl increase in serum creatinine from baseline within 2 days after surgery. Hepatic insufficiency was defined by a serum transaminase concentration > 120 U/L (3 times the upper limit of normal), bilirubin concentration > 3 mg/dL, or a diagnosis of cirrhosis.

### Statistical methods

The data analysis was performed using SPSS 24.0 statistical software (IBM Corp., Armonk, NY, USA). Continuous variables with normal distribution were presented as means ± standard deviations. Variables with skewed distribution data were presented as medians and interquartile ranges. Categorical variables were expressed as numbers and percentages. Continuous variables were compared using the t-test or Mann-Whitney U-test and categorical variables were compared using the chi-square test or Fisher’s exact probability method. The PaO_2_/FiO_2_ ratio at different time points was compared using the variance of repeated measurement data and the least significant difference (LSD) method was used for pairwise comparisons. *p* < 0.05 was considered statistically significant.

The propensity score matching (PSM) analysis was conducted using the R plug-in (PSM extension) in SPSS 24.0 (IBM Corp.), with a nearest-neighbor matching algorithm (1:3) and a caliper of 0.2. Propensity scores were generated using a multivariable logistic regression analysis model based on the following covariates: age, deep hypothermic circulatory arrest time, minimum temperature in CPB, and a PaO_2_/FiO_2_ ratio that met hypoxemia criteria.

## Results

Among 436 patients who underwent AADA surgery, 187 (42.9%) had hypoxemia and 43 were treated with low-dose iNO. After excluding coma (*n* = 5), cardiogenic shock (n = 5), and gastrointestinal ischemia (*n* = 1), 176 patients were included 40 and 136 in the iNO and control groups, respectively. Profound hypothermic circulatory arrest time and minimum CPB temperature were significantly different between the groups (Table 1). PSM was used to balance the baseline covariates. After PSM, patients were included in the iNO and control groups in a 1:3 ratio (Fig. 1). All reported parameters were balanced in both groups and no significant differences were detected (Table 2).

**Table 2:** Baseline characteristics of patients with AADA and hypoxemia at 24 h after surgery: post-PSM

Patients’ Characteristics	Control (*N* = 94)	iNO treatment (*N* = 40)	*p*-value
Age, y	50.5 ± 11.2	49.9 ± 11.2	0.763
Men	75 (79.8)	31 (77.5)	0.766
BMI	26.1(24.9, 28.6)	26.5(24.5, 27.7)	0.719
Comorbidity			
Hypertension	65 (69.1)	32 (80.0)	0.199
Diabetes	1 (1.1)	2 (5.0)	0.159
COPD	2 (2.1)	1 (2.5)	0.894
Hepatic insufficiency	1 (1.1)	1 (2.5)	0.530
Renal insufficiency	20 (21.3)	12 (30.0)	0.163
Cerebrovascular event	2 (2.1)	2 (5.0)	0.734
Tamponade	17 (18.1)	9 (22.5)	0.554
Myocardial infarction	8 (8.5)	2 (5.0)	0.479
Marfan Syndrome	3 (3.2)	3 (7.5)	0.270
Smoking	23 (24.5)	8 (20)	0.575
Laboratory test			
Leucocyte (10^9^/L)	11.8 ± 3.8	13.0 ± 3.3	0.086
Hemoglobin (g/L)	134.0(124.0, 143.0)	137.5(124.8, 150.3)	0.652
Platelet (10^9^/L)	162.0 (127.0, 212.0)	161.5(140.5, 200.0)	0.726
ALT (U/L)	27.0(20.0, 39.5)	30.5(19.0, 41.4)	0.934
Creatinine (μmol/L)	82.0(66.5, 108.2)	89.5(70.2, 132.0)	0.256
Troponin I (ng/ml)	0.02(0.01, 0.07)	0.02(0.01, 0.08)	0.489
PaO_2_ (mmHg)	83.5(70, 99)	80(67.7, 109.2)	0.132
Surgical procedure			
Total arch replacement	89 (94.7)	38 (95.0)	1.000
Hemi arch replacement	5 (5.3)	2 (5.0)	1.000
Concomitant CABG	9 (9.6)	2 (5.0)	0.377
CPB			
CPB time (min)	167.5(149.0, 183.0)	162.5(147.0, 191.5)	0.808
ACC time (min)	97.5(84.0, 109.3)	96.0(87.3, 117.3)	0.569
DHCA time (min)	26.0(20.8, 30.0)	24.0 (19.0, 28.0)	0.143
Minimum temperature in CPB (°C)	22.1(22.1, 24.3)	22.1(21.5, 23.5)	0.129
PaO_2_/FiO_2_ ratio meet hypoxemia criteria	109.0(85.2, 130.1)	95.0(82.9, 127.1)	0.524

*AADA* Acute type A aortic dissection, *PSM* Propensity score-matching, *iNO* Inhaled nitric oxide, *BMI* Body mass index, *COPD* Chronic obstructive pulmonary disease, *ALT* Alanine transaminase, *CPB* Cardiopulmonary bypass, *ACC* Aortic cross-clamp, *DHCA* Deep hypothermic circulatory arrest, *PaO*_*2*_ Partial pressure of oxygen, *FiO*_*2*_ Fraction of inspired oxygen

After PSM, the durations of mechanical ventilation and ICU stay were significantly shorter in the iNO group than in the control group (both *p* < 0.001). There were no significant differences in mortality, complications, or lengths of hospital stay. Eight patients (6.0%) died during the perioperative period, one in the iNO group due to sepsis, and 7 in the control group (2 from cardiac arrest and 5 from sepsis) (Table 3).

**Table 3:** Primary and secondary outcomes after PSM

Outcome	Control (*N* = 94)	iNO treatment (*N* = 40)	*p*-value
Primary outcome			
Mechanical ventilation time (hours)	69.0(47.8, 110.3)	39.0(31.2, 47.8)	< 0.001
ICU stay (hours)	179.5(114.0, 258.0)	122.0(80.8, 155.0)	< 0.001
Secondary outcome			
Inpatient deaths N (%)	7 (7.4)	1 (2.5)	0.479
In-hospital stay (days)	20.5(15.8, 28.5)	22.8(19.1, 27.5)	0.131
Postoperative drainage (24 h)	470(325, 750)	495(260, 667)	0.216
Peak CRP (mg/L)	7.1(2.4, 85.7)	8.0(2.6, 37.9)	0.676
Complication.			
Cardiac arrest	2 (2.1)	0 (0.0)	0.880
Re-intubation	7 (7.4)	2 (5.0)	0.888
Pneumonia	23 (24.5)	5 (12.5)	0.110
Hepatic insufficiency	20 (21.3)	4 (10.0)	0.119
AKI	55 (58.5)	19 (47.5)	0.241
CRRT	7 (7.4)	2 (5.0)	0.605
Paraplegia	1 (1.1)	0 (0.0)	0.513
Sepsis	6 (6.4)	4 (10.0)	0.466
Re-exploration for bleeding	2 (2.1)	2(5.0)	0.734
thrombocytopenia	28 (29.8)	13 (32.5)	0.755

*PSM* Propensity score-matching, *iNO* Inhaled nitric oxide, *ICU* Intensive care unit, *CRP* C-reactive protein, *AKI* Acute kidney injury, *CRRT* Continuous renal replacement therapy

**Fig. 2:**
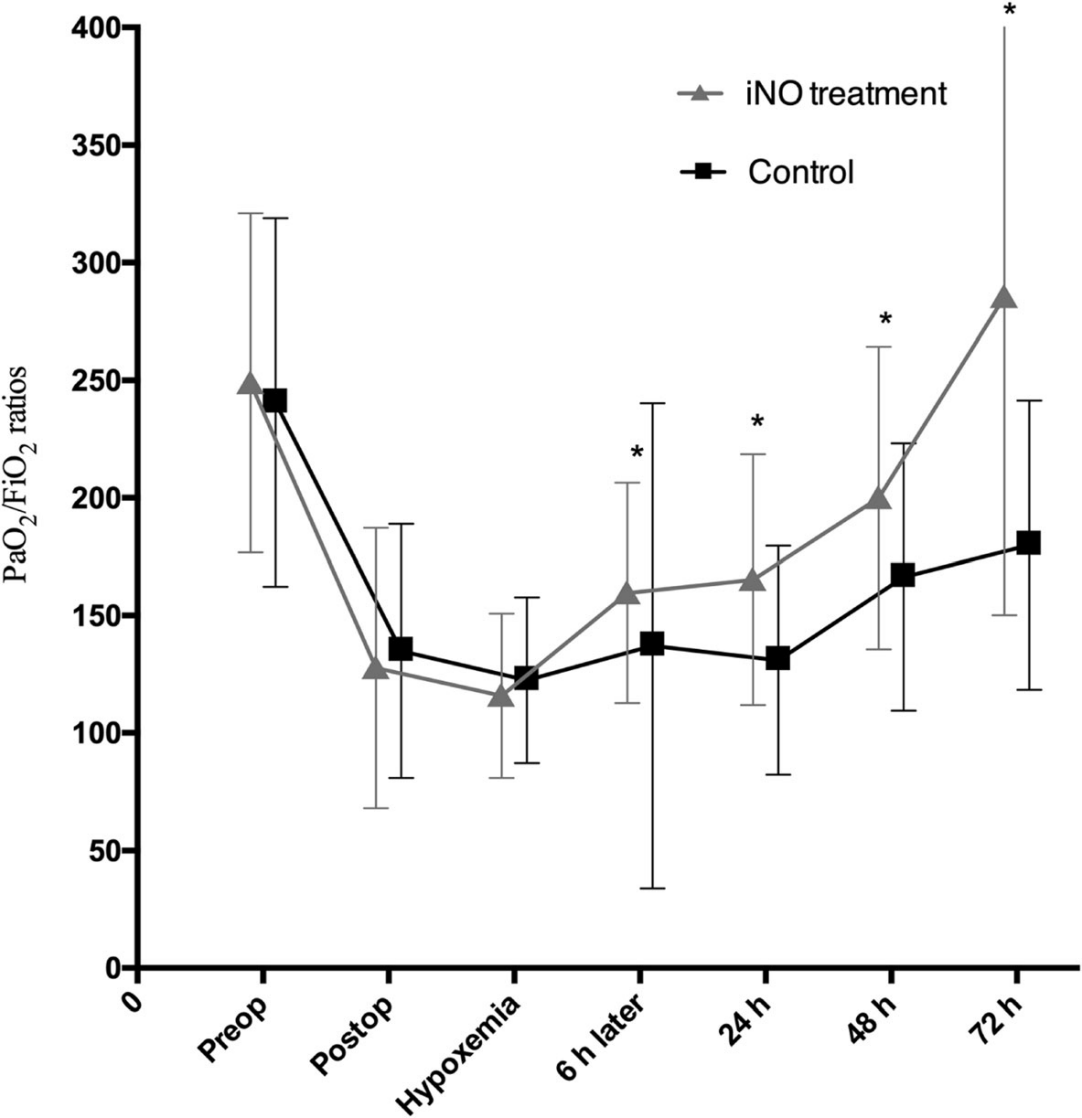
Comparison of perioperative PaO2/FiO2 ratios between the iNO and control groups. * *p* < 0.01 compared with oxygenation index before iNO. iNO: inhaled nitric oxide

There were no significant differences in the PaO_2_/FiO_2_ ratios between the groups preoperatively, upon postoperative ICU admission and at the first time hypoxemia criteria were met (*p* > 0.05). After administration of iNO for 6 h, the PaO_2_/FiO_2_ ratios in the iNO group were significantly higher than those in the control group (153.5 (125.8–186.2) vs. 123.1 (104.1–144.0); *p* < 0.001). The PaO_2_/FiO_2_ ratios in two groups improved with time, but the differences remained significant at 24 h (161.7 (125.4–197.3) vs. 125.3 (100.3–148.8); *p* < 0.001), 48 h (195.4 (147.3–253.1) vs. 156.8 (128.3–202.7); *p* = 0.004), and 72 h (258.8 (188.1–325.0) vs. 173.3 (134.4–220.2), *p* < 0.001) (Fig. 2).

We also compared all outcomes between the two groups without PSM as a sensitivity analysis and found shorter durations of mechanical ventilation and ICU stay and better PaO_2_/FiO_2_ ratios, which corresponded with the results after PSM (see Additional file 1).

The NO_2_ concentration was continuously monitored during iNO administration and was maintained at < 0.6 ppm. The MetHb levels in the iNO group were less than 1.5%. Platelet counts and drainage were comparable between the groups (*p* > 0.05) (Tables 3).

## Discussion

To the best of our knowledge, this is the first retrospective case-control study to explore the efficacy and safety of iNO for the treatment of postoperative hypoxemia among patients with AADA. In the present study, low-dose iNO improved patient oxygenation gradually over a 3-day period, decreased the duration of mechanical ventilation, and reduced the ICU length of stay. There were trends toward less postoperative mortality and pneumonia, but no significant differences were observed.

iNO rapidly led to ventilation/perfusion matching, then ameliorated oxygenation in the injured lungs. Rossaint et al. [14] first reported that iNO improved oxygenation in patients with ARDS in 1993. Subsequent studies showed similar improvements in oxygenation in a dose-dependent manner without better outcomes [10]. Multiple systematic reviews have shown that iNO does not reduce the mortality among patients with ARDS, nor does it shorten the duration of mechanical ventilation and it may even increase the incidence of renal impairment [9]. Nevertheless, one study showed that low iNO doses (< 5 ppm) improved lung function in ARDS survivors at 6 months [15]. Generally, iNO dilates blood vessels of ventilated alveoli and increases their blood flow, thereby counteracting the ventilation/perfusion mismatch [16]. Improvements in oxygenation occur rapidly, but the treatment does not practically resolve the disease. If the disease progresses, the alveoli will continue to collapse and the effects of iNO will disappear over several days. In our study, causes of lung injury, such as vessel rupture and CPB, were eliminated by the time the surgery was completed. This may be why the effects of iNO on oxygenation were obvious and sustained. Our results are similar to those of Prendergast et al. in their treatment of hypoxemia with iNO after coronary artery bypass grafting [17].

Another possible mechanism behind the beneficial outcomes after iNO is related to its anti-inflammatory effects. NO is involved in various physiological and pathological processes. It can be either protective or destructive in different conditions and according to the dose and time course [16, 18]. Studies in different animal models have demonstrated that iNO ameliorates lung injury by inhibiting inflammatory cytokines and oxidative damage [19–21]. A recent study on iNO-mediated prevention of bronchopulmonary dysplasia in Europe showed that iNO treatment may decrease several inflammatory and fibrotic factors in the lungs [22]. Preemptive iNO in human liver transplantation surgery led to clear anti-inflammatory effects in liver grafts which protected graft function, ameliorated pathological changes, and reduced postoperative morbidity [23]. In our study, we found no evidence of inflammation modulation since postoperative C-reactive protein levels were similar in the two groups. However, we speculate that the effects of iNO were associated with regulation of inflammation for several reasons: (1) systemic inflammation causes perioperative hypoxemia in AADA [24]; (2) treatments using systemic anti-inflammatory medicines such as glucocorticoid, sivelestat, and ulinastatin could play a role in attenuating hypoxemia [25, 26]. In our study, there was a trend toward lower inpatient mortality in the iNO treatment group, but this was not significant. Patients with AADA often were in critical condition and could have had complications other than lung injury.

Some ARDS studies indicated that iNO can adversely affect renal function [9]; however, the mechanism is unclear. A recent study showed that the administration of 80 ppm NO gas into the extracorporeal circulation in conjunction with postoperative iNO treatment significantly reduced the occurrence of renal dysfunction after valve replacement surgery [27]. Recently, Hyun-Su et al. reported that iNO did not worsen renal function after lung transplantation; nearly half of the patients in that study were administered extracorporeal membrane oxygenation [28]. In this study, the incidence of AKI and use of continuous renal replacement therapy (CRRT) were similar between the two groups.

During iNO treatment, routine MetHb and exhaled NO_2_ monitoring are required. With low-dose iNO, the concentrations of those two substances are small and stable; their safety has been confirmed in other studies of iNO in infants [22]. Another possible side-effect of iNO is that it can inhibit platelet aggregation and adhesion to the vascular endothelium, thereby prolonging bleeding time. We compared the number of patients with thrombocytopenia, daily pleural effusions, and those who underwent reoperations and found no significant between-group differences. However, active bleeding remained a contraindication to iNO. The price of iNO is about 1200 RMB per day (Chinese currency), which is equivalent to almost 174 USD per day and the average duration of iNO therapy was 3 days. The total in-hospital expenses of the two groups were similar (data not shown).

### Study limitations

Our study had several limitations. First, this was a retrospective study with a small sample size; therefore, potential biases could not be fully avoided. Because we did not calculate the required sample size before the study, the potential difference of outcomes may not be showed due to the underpower. A multicenter RCT with larger sample size would add more weight to these results. Second, we lacked long-term follow-up data on our patients. Thus, it is not clear how iNO affected the patients after discharge. Third, Selection of patients who had iNO remains elusive despite PSM and further prospective RCT is needed to identify patients who will benefit from iNO.

## Conclusion

This study showed that low-dose iNO treatment possibly improved pulmonary oxygenation and shortened the durations of mechanical ventilation and ICU stays among patients with hypoxemia after AADA. There were no clinical side-effects and no effects on postoperative morbidity and mortality. Therefore, further perspective multicenter trials to clarify the effect and mechanisms of iNO are necessary.

## Supplementary information

**Additional file 1.** Primary and secondary outcomes before PSM.

## Data Availability

All data have been retrieved from the institutional data base and are available from the corresponding author on reasonable request.

## Abbreviations

AADA: Acute type A aortic dissection
ABG: Arterial blood gases
ACC: Aortic cross-clamp
AKI: Acute kidney injury
ALT: Alanine transaminase
ARDS: Acute respiratory distress syndrome
BMI: Body mass index
CABG: Coronary artery bypass graft
COPD: Chronic obstructive pulmonary disease
CPB: Cardiopulmonary bypass
CRP: C-reactive protein
CRRT: Continuous renal replacement therapy
DHCA: Deep hypothermic circulatory arrest
eGFR: Estimated glomerular filtration rate
FiO2: Fraction of inspired oxygen
ICU: Intensive care unit
iNO: Inhaled nitric oxide
PaO2: Partial pressure of oxygen
PEEP: Positive end-expiratory pressure
PSM: Propensity score-matching
SIMV: Synchronized intermittent mandatory ventilation
