# Supplementary material for: Effects of inhaled nitric oxide for postoperative hypoxemia in acute type A aortic dissection: a retrospective observational study
Source: J Cardiothorac Surg. 2020 Jan 22;15:25. doi: 10.1186/s13019-020-1069-6 (PMC6977331; doi:10.1186/s13019-020-1069-6)
Supplement: Supplementary file 1 — Additional file 1. Primary and secondary outcomes before PSM. [file 13019_2020_1069_MOESM1_ESM.docx]

Dear editors:

Thank you for sparing your valuable time to review our work. Here we’d like to attach a supplement to compare with the outcomes we present in our article. This supplement is primary and secondary outcomes before propensity score-matching. According to data showed in the supplement, we found the durations of mechanical ventilation and ICU stay were significantly shorter in the iNO group than in the control group (both p<0.001). There were no significant differences in mortality, complications, and lengths of hospital stay. This finding is consistent with the result we showed in our article and large bias in our propensity score-matching analysis may be reasonably avoided.

We will be very glad to hear from you.

Yours sincerely.

Min Yu, MD

Department of Cardiovascular Surgery

Shanghai General Hospital

Shanghai Jiao Tong University School of Medicine

No. 100 Haining Road, Shanghai 200080, China

Email: [minyudr@163.com](mailto:minyudr@163.com)

Yang Liu, MD

Department of Cardiovascular Surgery

Changhai Hospital

Second Military Medical University

Shanghai, 200433, China

Tel.: +86 021-31161761

Fax: +86 021-65490979

Email: liuyang3722@163.com

|  | Supplement: Primary and secondary outcomes before PSM | | | |
| --- | --- | --- | --- | --- |
| Outcome | | Control  (N=136) | iNO treatment  (N=40) | p-value |
| Primary outcome | |  |  |  |
| Mechanical ventilation time (hours) | | 69.0 (47.0, 108.0) | 39.0 (31.3, 47.8) | <0.001 |
| ICU stay (hours) | | 161.0 (112.0, 254.0) | 122.0 (80.8, 155.0) | <0.001 |
| Secondary outcome | |  |  |  |
| Inpatient deaths N (%) | | 9 (6.6) | 1 (2.5) | 0.323 |
| In-hospital stay (days) | | 20.0 (16.0, 28.8) | 22.8 (19.1, 27.5) | 0.098 |
| Postoperative drainage (24 h) | | 510.0 (315.0, 745.5) | 495.0 (260.0, 667.5) | 0.214 |
| Peak CRP (mg/L) | | 7.0 (2.3, 82.6) | 8.0 (2.6, 38.0) | 0.560 |
| Complication. | |  |  |  |
| Cardiac arrest | | 2 (1.5) | 0 (0.0) | 1.000 |
| Re-intubation | | 10 (7.4) | 2 (5.0) | 0.871 |
| Pneumonia | | 32 (23.5) | 5 (12.5) | 0.132 |
| Hepatic insufficiency | | 24 (17.6) | 4 (10.0) | 0.359 |
| AKI | | 72 (52.9) | 19 (47.5) | 0.545 |
| CRRT | | 9 (6.6) | 2 (5.0) | 0.710 |
| Paraplegia | | 3 (2.2) | 0 (0.0) | 1.000 |
| Sepsis | | 9 (6.6) | 4 (10.0) | 0.708 |
| Re-exploration for bleeding | | 4 (2.9) | 2(5.0) | 0.892 |
| thrombocytopenia | | 41 (30.1) | 13 (32.5) | 0.777 |

PSM: propensity score-matching; iNO: inhaled nitric oxide; ICU: intensive care unit; CRP: c-reactive protein; AKI: acute kidney injury; CRRT: continuous renal replacement therapy;
